# S182. CHANGE IN CORTICAL MORPHOMETRY IN INDIVIDUALS WITH PERSISTING PSYCHOTIC EXPERIENCES: A LONGITUDINAL PILOT STUDY

**DOI:** 10.1093/schbul/sby018.969

**Published:** 2018-04-01

**Authors:** Leon Fonville, Mark Drakesmith, Avi Reichenberg, Derek Jones, David Linden, Anthony David

**Affiliations:** 1 University of Westminster; 2 Cardiff University Brain Research Imaging Centre;; 3 Icahn School of Medicine at Mount Sinai; 4 MRC Centre for Neuropsychiatric Genetics & Genomics, Cardiff University School of Medicine; 5 Institute of Psychiatry, King’s College London

## Abstract

**Background:**

There is an increasing interest in the presence of psychotic symptoms in the general population that do not meet the threshold for psychotic disorder. Such psychotic experiences (PEs) are more prevalent than conditions such as schizophrenia and often manifest during childhood and adolescence. PEs are a risk factor for psychotic disorders and a range of adverse psychosocial outcomes. PEs can become abnormally persistent with a corresponding increase in psychiatric morbidity. Previous research has highlighted subtle deviations in brain structure associated with the presence and persistence of PEs, but longitudinal assessments are needed to gauge if these deviations continue, perhaps as part of an atypical neurodevelopmental trajectory.

**Methods:**

Longitudinal imaging data, taken at ages 20 and 25, were available for 25 young adults who were part an MRI nested case control study within the Avon Longitudinal Study of Parents and Children. They were selected originally on the basis of presence or absence of PEs. Cortical surface data were analysed using Freesurfer. Presence of PEs was assessed at ages 18, 20, and 25 and we defined persistence as endorsing PEs at multiple time-points. Average cortical volume and thickness were extracted from each brain parcellation. We additionally calculated fractal dimensionality (FD) of each parcellation using a box-count algorithm to capture shape complexity. We compared the rate of change between healthy controls (HC) and those with persistent PEs in each parcellation and used permutation testing to control for multiple comparisons.

**Results:**

Both HC and PEs showed the expected age-related net loss in brain volume; an increase in white matter volume offset by a greater reduction in grey matter. We identified greater volume loss in PEs in the left parietal lobe and further examination of local volume highlighted additional changes. PEs were associated with a greater rate of volume loss in the anterior cingulate, postcentral, and lingual gyrus in the left hemisphere and in the right inferior parietal lobule and pars orbitalis. Thinning of the left inferior parietal lobule was greater in those with PEs. There was further converging evidence of focal abnormalities in the left postcentral gyrus in terms of reductions in cortical thickness and FD in PEs. Similarly, we found reduced FD relative to HC in the left rostral anterior cingulate. There was additional evidence for reductions in FD in the left hemisphere in the cuneus, isthmus cingulate, and middle frontal gyrus.

**Discussion:**

Our findings highlight a deviation from typical age-related changes in brain volume in individuals with persisting manifestations of PEs. Though these changes could reflect an acceleration of the typical volume loss that is seen with aging, there are several points of evidence against this. We found no differences in global volume changes and only the left parietal lobule was found to show a greater volume loss in PEs. On a local scale, findings seemed to mostly converge on parietal and cingulate regions in the left hemisphere with some evidence of aberrations in frontal regions. These pilot data are, uniquely, unconfounded by illness and treatment related factors and highlight the continued need for longitudinal assessments of brain structure in relation to PEs; there is an increasing risk of transitioning to a psychotic disorder with persistence of PEs and our findings may reflect the neuroanatomical basis for an anomalous developmental trajectory related to psychotic disorders.

